# Supplementary material for: Towards unified AI-driven fracture mechanics: the extended deep energy method (XDEM)
Source: Nat Commun. 2026 Aug 15;17:8492. doi: 10.1038/s41467-026-76748-1 (PMC13484471; doi:10.1038/s41467-026-76748-1)
Supplement: Supplementary file 3 [file 41467_2026_76748_MOESM3_ESM.zip › pic/Results/Stress_intensity_factor/mode3/crack3.pdf]

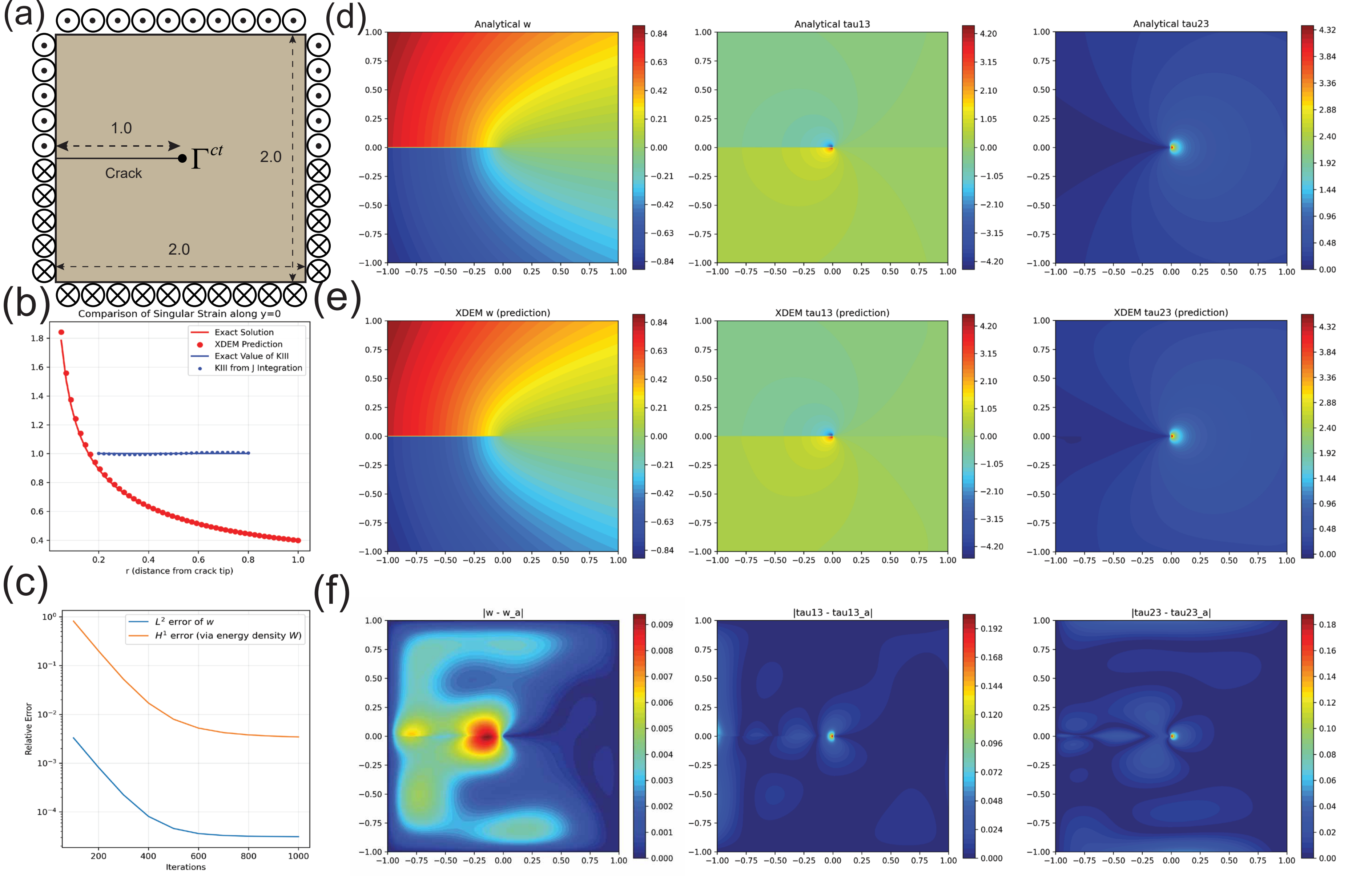

(a)
1.0
Crack
Γ^{ct}
2.0
2.0
(b)
Comparison of Singular Strain along y=0
Exact Solution
XDEM Prediction
Exact Value of KIII
KIII from J Integration
r (distance from crack tip)
(c)
L^2 error of w
H^1 error (via energy density W)
Relative Error
Iterations
(d)
Analytical w
Analytical tau13
Analytical tau23
(e)
XDEM w (prediction)
XDEM tau13 (prediction)
XDEM tau23 (prediction)
(f)
|w - w_a|
|tau13 - tau13_a|
|tau23 - tau23_a|
